# Immunosenescence-Related Transcriptomic and Immunologic Changes in Older Individuals Following Influenza Vaccination

**DOI:** 10.3389/fimmu.2016.00450

**Published:** 2016-11-02

**Authors:** Richard B. Kennedy, Inna G. Ovsyannikova, Iana H. Haralambieva, Ann L. Oberg, Michael T. Zimmermann, Diane E. Grill, Gregory A. Poland

**Affiliations:** ^1^Mayo Clinic Vaccine Research Group, Department of General Internal Medicine, Mayo Clinic, Rochester, MN, USA; ^2^Department of Health Sciences Research, Division of Biomedical Statistics and Informatics, Mayo Clinic, Rochester, MN, USA

**Keywords:** aging, DNA methylation, gene expression profiling, immunity, influenza A/H1N1 virus, influenza vaccines, miRNA

## Abstract

The goal of annual influenza vaccination is to reduce mortality and morbidity associated with this disease through the generation of protective immune responses. The objective of the current study was to examine markers of immunosenescence and identify immunosenescence-related differences in gene expression, gene regulation, cytokine secretion, and immunologic changes in an older study population receiving seasonal influenza A/H1N1 vaccination. Surprisingly, prior studies in this cohort revealed weak correlations between immunosenescence markers and humoral immune response to vaccination. In this report, we further examined the relationship of each immunosenescence marker (age, T cell receptor excision circle frequency, telomerase expression, percentage of CD28^−^ CD4^+^ T cells, percentage of CD28^−^ CD8^+^ T cells, and the CD4/CD8 T cell ratio) with additional markers of immune response (serum cytokine and chemokine expression) and measures of gene expression and/or regulation. Many of the immunosenescence markers indeed correlated with distinct sets of individual DNA methylation sites, miRNA expression levels, mRNA expression levels, serum cytokines, and leukocyte subsets. However, when the individual immunosenescence markers were grouped by pathways or functional terms, several shared biological functions were identified: antigen processing and presentation pathways, MAPK, mTOR, TCR, BCR, and calcium signaling pathways, as well as key cellular metabolic, proliferation and survival activities. Furthermore, the percent of CD4^+^ and/or CD8^+^ T cells lacking CD28 expression also correlated with miRNAs regulating clusters of genes known to be involved in viral infection. Integrated (DNA methylation, mRNA, miRNA, and protein levels) network biology analysis of immunosenescence-related pathways and genesets identified both known pathways (e.g., chemokine signaling, CTL, and NK cell activity), as well as a gene expression module not previously annotated with a known function. These results may improve our ability to predict immune responses to influenza and aid in new vaccine development, and highlight the need for additional studies to better define and characterize immunosenescence.

## Introduction

Aging is associated with a variety of physiological changes, including a decline in immune function termed “immunosenescence.” Although immunosenescence is associated with aging, it is a multifactorial phenomenon characterized by dysregulated immune function at multiple levels and among diverse cell subsets. Adaptive immunity is affected as naïve T cell production declines, functionally inert memory cells accumulate, lymphocyte signaling is altered, and B cells and plasma cells decline in number and lose functional capacity (1–5). As a result of these and other changes, older individuals are more susceptible to influenza, have higher mortality rates, have more severe clinical outcomes, and have a decreased ability to respond to vaccination.

The 2009 H1N1 pandemic led to approximately 12,000 deaths in the U.S. and >90% of those who died were >65 years old (2, 6), a trend also seen with other influenza strains (7). A primary factor in the increased susceptibility to influenza is immunosenescence. In this age group, influenza vaccines are estimated to be <50% effective against influenza (8).

Modern vaccine development must recognize the needs of the elderly (in the U.S., this age group is expected to double in size by 2050) (9) and develop vaccine formulations that specifically overcome immunosenescence-related defects in order to elicit robust, reliable, protective immunity. Results of these efforts include high-dose vaccines to improve antigen presentation; the use of adjuvants (MF59, AS03) to enhance immunogenicity; mucosal delivery systems to induce localized immune responses at the site of entry; and intradermal delivery to deposit antigen in areas rich with antigen-presenting cells (APCs) (10–13).

A necessary precursor to improving vaccines for older individuals is to better understand the fundamental mechanisms underlying immunosenescence. We have previously published on the impact of specific immunosenescence markers on influenza vaccine-specific humoral response (14). In this report, we seek to extend our understanding of common markers of immunosenescence by examining their impact on the transcriptome, DNA methylation, miRNA, and the proteome prior to and following receipt of the seasonal trivalent-inactivated influenza vaccine (TIV).

We have previously reported on the associations between markers of immunosenescence and humoral immune responses to influenza vaccination in this cohort (14). The availability of high-dimensional datasets (DNA methylation, mRNA expression, miRNA expression, and shotgun proteomics) provided a unique opportunity to evaluate the effect of immunosenescence on the biological processes that lead to immune response. Our data indicate that, while each marker was associated with different genes, miRNAs, proteins, or CpG sites, the variables were all associated with a similar set of biological pathways and functional genesets. Our results also indicate that dysregulated immune function due to immunosenescence may be symptomatic of larger effects on underlying biological processes, such as cell proliferation, regulation of the actin cytoskeleton, cell signaling, and a variety of metabolic activities. Our data also suggest that these effects come about through alterations in gene regulation (DNA methylation and miRNA production) and expression. For example, we identified multiple CpG sites in KLF14 whose methylation pattern was associated with age; which may contribute to differential TGFβ signaling in older adults.

## Materials and Methods

### Subject Recruitment

As previously reported, 159 subjects in good general health, between the ages of 50 and 74 (inclusive), were recruited at the Mayo Clinic in Rochester, MN, USA (15). Each recipient was vaccinated with the 2010–2011 inactivated influenza vaccine containing A/California/7/2009 H1N1-like, A/Perth/16/2009 H3N2-like, and B/Brisbane/60/2008-like viruses. Blood was drawn from each subject before (day 0) and after (days 3 and 28) immunization. Informed consent was obtained from each subject upon enrollment and prior to any study interventions. This study was approved by Mayo Clinic’s Institutional Review Board (IRB). All experiments involving influenza virus were carried out in BSL-2 certified biosafety hoods by laboratory personnel who receive yearly influenza vaccination and have completed mandatory institutional biosafety training.

### Assessment of Telomerase and T Cell Receptor Excision Circles

TERT mRNA expression was measured using quantitative real-time PCR, with minor modifications to the previously described method (16). The relative abundance of each transcript was calculated using their respective standard curves. The normalized results are expressed as the ratio of TERT to GAPDH expression. Quantitative real-time PCR for sjTREC was performed as previously described (14, 17). Standard curves were utilized to calculate the ratio of T cell receptor excision circle (TREC) copies versus CCR5 copies.

### Influenza Humoral Immunity Measurements

Humoral immunity to influenza A/H1N1was measured by multiple methods using the influenza A/California/07/2009 (H1N1)-like virus strain. The hemagglutination inhibition assay (HAI) was performed as this is the gold-standard correlate of protection for influenza. The virus neutralization assay (VNA) was performed in order to characterize a distinct functional outcome also associated with protection. We also performed a memory B cell ELISPOT, as previously described (14, 18, 19), in order to quantitate influenza-specific B cell numbers in each subject. The memory B cell ELISPOT assay provides a means of evaluating immunologic memory and provides complementary information to the titers of influenza-specific Abs present in each subject’s serum. Collectively, these immune outcomes provide a more comprehensive characterization of each subject’s humoral immune status before and after vaccination.

### Next-Generation Sequencing mRNA and miRNA Expression

The next-generation sequencing (NGS) methods are identical to those previously published (20–24). Briefly, total RNA was extracted (RNeasy Plus mini Kit, Qiagen) from cryopreserved PBMCs, RNA quantity and quality was assessed, poly-A RNA was isolated using olido-dT coated beads, fragmented, reverse transcribed into cDNA, and combined with Illumina adaptor sequences. After validation, cDNA libraries were sequenced on an Illumina HiSeq 2000 (Illumina; San Diego, CA, USA) with Illumina’s Single Read Cluster Generation kit (v2) and 50 Cycle Illumina Sequencing Kit (v3). The sequencing reads were aligned to build 37.1 of the human genome using TopHat (1.3.3), and Bowtie (0.12.7). Gene counts were performed using HTSeq (0.5.3p3), while BEDTools (2.7.1) was used to map normalized read count to individual exons (25–28).

### DNA Methylation

DNA methylation methods are identical to those previously described (29). Briefly, subject DNA was isolated, bisulfite modified, and genome-wide DNA methylation patterns were assessed using the Illumina’s HumanMethylation450 BeadChip and specimens were allocated to bead chips such that gender and timepoints were balanced over bead chips. In addition to standard laboratory checks, data quality was assessed via Minus versus Average (MVA) plots. The data were normalized via the following steps: (1) color-bias adjustment; (2) quantile normalization of intensity values between different flow cells, within probe design; and (3) beta-mixture quantile normalization (BMIQ) (30–32).

### Proteomic Analysis

PBS-washed PBMC samples were subjected to tryptic digestion and lysis. The protein concentration in each sample was quantitated by BCA assay against a bovine serum albumin (BSA) standard. A total amount of 80 µg protein was used for the peptide fractionation and LC-MS/MS analysis. SAX (strong anion exchange) fractionation was performed with stepped elutions of decreasing pH volatile buffers using volatile buffer kit (Column Technology, Inc., Fremont, CA, USA) on disposable pipette tips packed with Poros strong anion exchange phase (TT2PSA, Glygen). Ten micrograms of digest from each sample were distributed among the six SAX fractions. Protein level data were normalized on the log_2_ scale via a two-stage iterative ANOVA that iterates between estimating the sample effects and the protein effects (33). This iterative method is necessary in order to account for the data-dependent missingness.

### Cellular Immunophenotyping by Flow Cytometry

Cryopreserved PBMCs were thawed, stained with fluorochrome-conjugated antibodies and analyzed, as previously described (14). Two separate flow cytometry panels were used to phenotype immune cells. For the innate/APC panel, we used CD-11c-V450, CD3-V500, CD86-FITC, CD-56-PE, CD123-APC, CD20-PerCP-Cy5.5, HLA-DR-Alexa 700, CD16-PE-Cy7, and CD14-APC-Cy7. The Treg panel utilized: CD28-BV 421, HLA-DR-V500, CD25-BV 605, CD45RO-FITC, CD38-PE, CD194-PE-Cy7, CD127-AF647, CD4-AF700, and CD3-APC-Cy7. All antibodies were purchased from BD Biosciences, San Jose, CA, USA or eBioscience, San Diego, CA, USA. FlowJo (Treestar Inc.) v10 was utilized for gating specific cell subsets, surface expression of CD28, and calculating the CD4/CD8 ratio.

### Statistical Analyses

Differences in male and female expression of the immunosenescence markers were assessed by the Wilcoxon rank-sum test. Associations between all data types and measures of chronological or biological measures of age were assessed via Spearman’s non-parametric correlation. False discovery rate *q*-values were computed using the Storey and Tibshirani method (34) when comparing with high-dimensional data. For all correlation analyses, we set significance and false discovery thresholds of *p* ≤ 0.001 and *q* ≤ 0.15. False discovery rates for the biologic function analysis were calculated using the method of Benjamini and Hochberg (35).

Penalized elastic net regression was used to build regression models with the goal of understanding the biological processes that explain variation in vaccine response. The response (dependent) variable was day 28 HAI titer on the log2 scale. Covariates considered in each of the models were the genes from the modules defined by Chaussabel et al. (36).^1^ Immunosenescence markers were allowed to compete with gene expression variables. Gender was included in all models in order to increase precision. Since the genes considered as covariates were chosen agnostic to the endpoint, this particular step was not cross validated. In determining genes to add to the model, 10-fold cross validation was used to determine the optimal number to add based on the minimum cross-validated mean squared error (MSE). The “glmnet” function in R was used for model building (37). The tuning parameter alpha was set to 0.9, reflective of the elastic net penalty (a combination of the L1 LASSO and L2 ridge penalties). Results are presented with cross-validated MSE and standardized coefficients.

### Network Biology

Multiple resources were combined [PID (38), HPRD (39), CCSB (40), the 7.8% of STRING (41) with confidence score ≥70%, and HumanNet (42)] to create a network biology framework with which to analyze the datasets in this study. Visualization was performed in Cytoscape (43) version 3.2.1. Pathway enrichment was performed using 138 pathway definitions (out of 186: see Table S7 in Supplementary Material) downloaded from MSigDB’s index of KEGG canonical pathways (44, 45), having removed disease associations pathways that are derived from combinations of other pathways.

## Results

Our cohort consisted of 159 older (61.6% female; 50–74 years of age) recipients of the 2010–2011 TIV. We assessed the following markers of immunosenescence prior to vaccination: chronological age, TREC frequency, telomerase expression (TERT), the percentage CD4^+^ and CD8^+^ T cells that were CD28 low (%CD4^+^CD28^−^ and %CD8^+^CD28^−^), and the CD4^+^/CD8^+^ ratio. The distributions of both immunosenescence markers and immune response outcomes in our cohort are illustrated in Table 1 and Figure S1 in Supplementary Material. Female study subjects had significantly higher TREC levels (*p* = 0.0003) and an increased CD4^+^/CD8^+^ ratio (*p* = 0.04) (Table 1). Correlations among these immunosenescence markers were weak, with two exceptions: a negative correlation, *r* = −0.33, between TREC and the %CD8^+^CD28^−^ cells; and a positive correlation, *r* = 0.49, between the %CD4^+^CD28^−^ cells and the %CD8^+^CD28^−^ cells.

**Table 1:** **Distribution of immunosenescence and immunologic markers**.

Immunosenescence markers			
Marker	Median (males)	Median (females)	*p*-Value^a^
Age (years)	59.7	59.4	0.9
TREC (copy number of TREC/GAPDH)	288.7	608.0	0.0003
TERT (copy number of TERT/CCR5)	0.000021	0.000028	0.1
% CD4^+^CD28^−^ cells	1.7	2.3	0.2
% CD8^+^CD28^−^ cells	39.1	35.9	0.5
CD4^+^/CD8^+^ Ratio	3.95	4.96	0.04

**Immunologic markers**			

**Marker**	**Median titer or SFC**	**Range**	

HAI	1:80	<1:10–1:1280	
VNA	1:80	<1:10–1:2560	
B cell ELISPOT	10.5	0.0–84.5	

**Serum cytokines and chemokines**			

**Cytokine/chemokine**	**Median (pg/ml)**	**IQR**	

IFNγ	0.35	0.12–0.69	
IFNα-2a	0.11	0.02–0.24	
IL-1a	0.00	0.00–0.00	
IL-1b	0.02	0.00–0.03	
IL-2	0.09	0.04–0.13	
IL-4	0.01	0.00–0.06	
IL-5	0.24	0.15–0.40	
IL-6	0.41	0.27–0.67	
IL-7	3.90	2.73–5.36	
IL-8	4.98	3.48–7.56	
IL-10	0.86	0.64–1.25	
IL-12p70	0.08	0.00–0.25	
IL-13	0.12	0.02–0.27	
GM-CSF	0.17	0.05–0.404	
TNFα	5.02	4.02–6.48	
Eotaxin	237.80	180.90–321.01	
Eotaxin-3	22.65	12.89–42.41	
MIP-1b	127.01	90.97–172.30	
TARC	420.81	264.00–646.53	
IP-10	144.94	101.43–216.89	
MCP-1	427.13	340.48–548.81	
MDC	253.95	190.40–375.07	
MCP-4	727.37	523.97–1,049.84	
RANTES	110,980.93	80,695.69–155,186.77	
MIP-1a	1.02	0.41–2.05	

*^a^p-values determined as described in the Sections from “Materials and Methods” to “Statistical Analyses*.”

*IQR, interquartile range*.

### Multivariate Modeling of Immunosenescence and Vaccine Response

**Figure 1:**
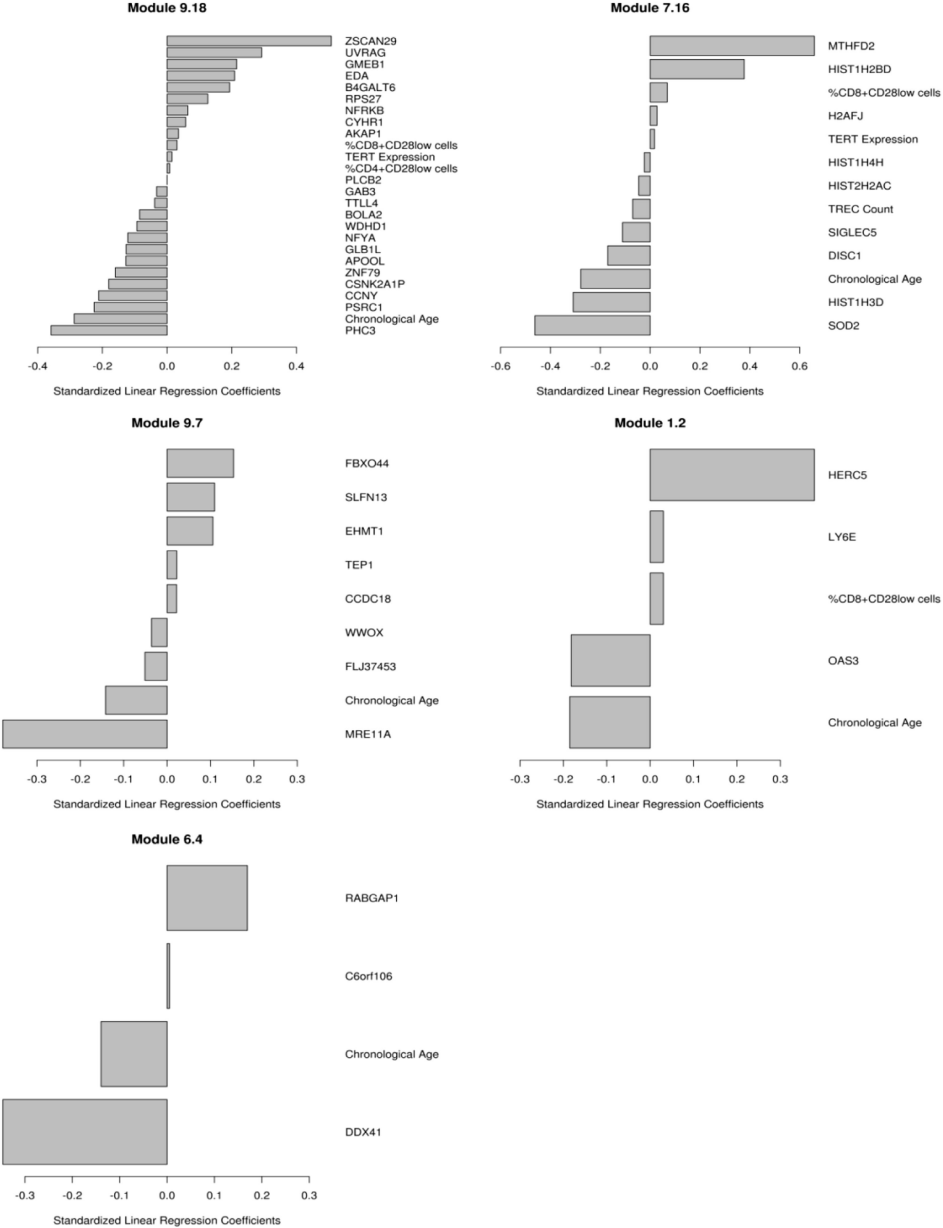
**Multivariate correlates of HAI response**. Results from the penalized elastic net regression models: baseline gene expression from the genes in the models and the immunosenescence markers were included as competing covariates. Results are presented as standardized coefficients for the variables that remained in each of the modules. Each model is described in the text.

We have previously published on the moderate correlations between markers of immunosenescence and influenza A/H1N1-specific humoral immune responses (HAI, VNA, B cell ELISPOT) (14). We utilized transcriptional modules, defined by Chaussabel et al. (36), as a variable reduction strategy to avoid both overfitting and overwhelming the model with too many parameters/genes. We developed penalized regression models for predicting humoral immune response to TIV, as measured by HAI, that included gene expression and the immunosenescence markers. The use of these modules further facilitates data interpretation, since many of the modules have defined immunologic functions (46). The data in Table 2 and Figure 1 extend our previous work to include all of our measured immunosenescence markers in the framework of the transcriptional modules. These results indicate that while immunosenescence is associated with HAI response, it explains only a small portion of the variation in HAI. As our study cohort had high-dimensional data covering multiple biological spaces (epigenome, miRNome, transcriptome, proteome), we conducted further analyses (described below) in order to uncover additional biological functions associated with each immunosenescence markers.

**Table 2:** **Penalized regression modeling of day 28 HAI response**.

Baseline Gene Expression (Day 0) and Immunosenescence Markers			
Module	Functional Activity	MSE	Model-Specific Elements
M9.20	Undetermined	2.40	MRE11A, LYZ, ITGA1, **Chronological Age**, ERCC8, KCTD3, THUMPD3, SAMD4A, SPON2
M6.4	Undetermined	2.41	DDX41, **Chronological Age**, C6orf106, RABGAP1
M9.7	Undetermined	2.43	MRE11A, **Chronological Age**, FLJ37453, WWOX, CCDC18, TEP1, EHMT1, SLFN13, FBXO44
M5.15	Neutrophils	2.51	BPI, **Chronological Age**
M9.18	Undetermined	2.51	PHC3, **Chronological Age**, PSRC1, CCNY, CSNK2A1P, ZNF79, APOOL, GLB1L, NFYA, WDHD1, BOLA2, TTLL4, GAB3, PLCB2, **%CD4^+^CD28**^−^ **cells, TERT Expression, %CD8^+^CD28**^−^ **cells**, AKAP1, CYHR1, NFRKB, RPS27, B4GALT6, EDA, GMEB1, UVRAG, ZSCAN29
M1.2	Interferon	2.52	OAS3, **Chronological Age**, LY6E, **%CD8^+^CD28**^−^ **cells**, HERC5
M7.16	Undetermined	2.53	SOD2, HIST1H3D, **Chronological Age**, DISC1, SIGLEC5, **TREC Count**, HIST2H2AC, HIST1H4H, **TERT Expression**, H2AFJ, **%CD8^+^CD28**^−^ **cells**, HIST1H2BD, MTHFD2
M7.15	Undetermined	2.55	SLC22A18, **Chronological Age**
M8.58	Undetermined	2.55	**Chronological Age**, MTUS1, LPHN1, SLC25A26
M5.12	Interferon	2.55	**Chronological Age**, RBCK1, TAP2

*Gene Modules obtained from: http://www.biir.net/public_wikis/module_annotation/G2_Trial_8_Modules. MSE, mean squared error*.

*Immunosenescence markers are in bold font*.

### DNA Methylation

In our study cohort, DNA methylation at baseline exhibited multiple, strong correlations with each immunosenescence marker (Table S1 in Supplementary Material). Seventy CpG sites were associated with age; 238 CpG sites with TERT; while over 693 CpG sites were associated with TREC, Over 1,000 CpG sites were associated with each of the following markers: the percent of CD28^−^ CD4^+^ T cells, the percent of CD28^−^CD8^+^ T cells, or the CD4^+^/CD8^+^ T cell ratio.

### miRNA Expression

Fifty-three subjects had miRNA data available. Using this dataset, we identified a number of immunosenescence-associated miRNAs (Table S2 in Supplementary Material). Baseline levels of several miRNAs exhibited correlations with multiple immunosenescence markers: miR-7-5p, miR-9-5p, and miR-196a-5p (correlated with the %CD4^+^CD28^−w^ and the %CD8^+^CD28^−w^); miR-125b-5p and miR-335-5p (correlated with TREC and the %CD8^+^CD28^−^). Pathway enrichment analysis on the predicted gene targets of the immunosenescence-correlated miRNAs (Table S3 in Supplementary Material) indicated that immunosenescence markers were associated with signaling pathways, T and B cell activation pathways, hormone and growth factor signaling, and metabolic functions.

### mRNA Expression

We examined correlations between baseline gene expression patterns and immunosenescence markers (Table S4 in Supplementary Material) using the same statistical cut-offs (*p* ≤ 0.001 and *q* ≤ 0.15). In our dataset, only one gene met the threshold for correlation with age: *ROBO1* (*p* = 5.15 × 10^−6^; *q* = 0.07). By contrast, a varying number of genes had expression levels correlated with the other markers of immunosenescence: 117 genes with TREC; 2,036 with TERT; 386 genes with the %CD8^+^CD28^−^ cells; 611 genes with the %CD4^+^CD28^−w^ cells; and 801 genes correlated with CD4^+^/CD8^+^ ratio. As expected, there was a high degree of overlap between the genes correlated with the two CD28^−^ T cell subsets.

### Protein Expression

PBMC samples from a subset of our cohort (*n* = 50) underwent shotgun proteomics analysis. Correlations between immunosenescence markers and baseline protein expression levels are listed in Table S5 in Supplementary Material. TREC levels exhibited a negative correlation with the vitamin receptor genes RXRA and RXRG, while the percentage of CD28^−^CD4^+^ T cells was highly correlated with GZMH2, CD2, and genes encoding IFN-inducible proteins: PYHIN1 and GBP1. There were no proteins correlated with TERT or the percentage of CD8^+^CD28^−^ T cells at the established significance threshold.

### Immunophenotyping

We performed three separate immunophenotyping panels in order to quantitate: major leukocyte populations (NK cells, monocytes, DCs, B cell subsets, T cell subsets), as well as HLA and CD86 expression on the APCs. Baseline cell subsets and phenotypes correlated with each marker of immunosenescence are included in Table S6 in Supplementary Material. We identified negative correlations between TREC levels for the myeloid DC frequency. The CD4/CD8 ratio was also negatively correlated with the % of CD127^+^ memory T helper cells, and the percentage of NK T cells.

### Assessment of Biological Function

In order to develop a better understanding of these processes, we assessed the biological functions associated with the CpG sites, miRNAs, genes, and proteins correlated with each immunosenescence marker. The DIANA tool^2^ was used to determine the KEGG pathways controlled by the predicted targets of the immunosenescence-correlated miRNAs. The CpG sites, miRNA, and protein data were each mapped to their respective gene symbols and immunosenescence-correlated variables were analyzed using the DAVID functional gene annotation tool^3^ (47, 48). Chronological age, despite serving as the *de facto* “driver” of immunosenescence, was the marker with the fewest significant associations at the “per-variable” level and the DAVID and DIANA tools were unable to assign biological function to the limited number of associated variables. Telomerase activity is an indicator of cellular senescence and, in contrast to age, was correlated with a number of transcripts enriched in the lysosome pathway (Table 3) while TERT-correlated miRNAs were predicted to control a variety of signaling, metabolic, cytoskeletal, developmental, and cell proliferation processes. T cell receptor excision circles are remnants of the T cell receptor rearrangements that occur during the development of naïve T cells. At the miRNA level, we identified a large number of TREC-correlated pathways involved in cellular signaling, proteolytic activity, metabolism, cellular developmental, cell adhesion, and cell communication (Table 4). The biological functions attributed to variables correlating with the percentage of CD28^−^ T cells (both CD4 and CD8) are listed in Tables 5 and 6 and displayed a large amount of overlap, particularly in the miRNA-controlled pathways where 70 of the 74 processes correlated with the percentage of CD8^+^ CD28^−^ T cells are also correlated with the percentage of CD28^−^ CD4^+^ T cells. The CD4^+^/CD8^+^ T cell ratio has also been identified as a marker of aging, immunosenescence, and increased mortality (49, 50). Our data (Table 7) indicated that pathways primarily involved in immune function (NK cell mediated cytotoxicity, T cell receptor signaling, Fc gamma receptor-mediated phagocytosis, phosphatidylinositol signaling, leukocyte transmigration) and tumorigenesis were correlated with the CD4/CD8 T cell ratio.

**Table 3:** **Biological functions correlated with TERT**.

	Biological function or pathway	Fold enrichment	*p*-Value^1^	Benjamini *q*-value^1^
CpG	None identified			
mRNA	Lysosome	3.3	2.3E–12	4.2E–10
Protein	None identified			

	**Pathway**	***p*-Value^1^**	**# Genes**	**# miRNAs**

miRNA	Endometrial cancer	0.00064	4	1
	Regulation of actin cytoskeleton	0.00070	8	1
	p53 signaling pathway	0.0016	4	1
	Glioma	0.0016	4	1
	ErbB signaling pathway	0.0032	4	1
	d-Glutamine and d-glutamate metabolism	0.0053	1	1
	Acute myeloid leukemia	0.011	3	1
	Neurotrophin signaling pathway	0.012	5	1
	Small cell lung cancer	0.012	4	1
	GnRH signaling pathway	0.017	4	1
	Prostate cancer	0.017	4	1
	Dorso-ventral axis formation	0.022	2	1
	Thyroid cancer	0.022	2	1
	PI3K–Akt signaling pathway	0.031	8	1
	mTOR signaling pathway	0.037	3	1
	MAPK signaling pathway	0.043	7	1

*^1^*p*-values and *q*-values were determined by the DIANA (miRNA data) and DAVID (all other data types) tools as described in the Sections from “Materials and Methods” to “Assessment of Biological Function*.”

**Table 4:** **Biological functions correlated with TREC**.

	Biological function or pathway	Fold enrichment	*p*-Value^1^	Benjamini *q*-value^1^
CpG	None identified			
mRNA	None identified			
Protein	None identified			

	**Pathway**	***p*-Value^1^**	**# Genes**	**# miRNAs**

miRNA	MAPK signaling pathway	6.22E−10	40	5
	Neurotrophin signaling pathway	2.02E−06	20	5
	Ubiquitin-mediated proteolysis	6.49E−05	21	5
	d-Glutamine and d-glutamate metabolism	0.00035	2	2
	GnRH signaling pathway	0.00038	14	5
	Insulin signaling pathway	0.0020	18	5
	RNA transport	0.0020	18	6
	Long-term potentiation	0.0025	11	5
	PI3K–Akt signaling pathway	0.0047	35	5
	Calcium signaling pathway	0.0054	21	5
	Small cell lung cancer	0.0069	12	4
	Dilated cardiomyopathy	0.011	12	5
	T cell receptor signaling pathway	0.012	14	4
	Hypertrophic cardiomyopathy (HCM)	0.016	11	5
	p53 signaling pathway	0.024	9	4
	Hepatitis B	0.038	16	4
	Drug metabolism – cytochrome P450	0.039	3	2
	Histidine metabolism	0.039	5	3
	Amyotrophic lateral sclerosis (ALS)	0.039	8	3
	B cell receptor signaling pathway	0.039	10	4
	Acute myeloid leukemia	0.039	8	4
	Valine, leucine, and isoleucine biosynthesis	0.042	1	1
	Gap junction	0.042	12	4
	Arrhythmogenic right ventricular cardiomyopathy (ARVC)	0.043	12	5
	Chemokine signaling pathway	0.044	19	5

*^1^*p*-values and *q*-values were determined by the DIANA (miRNA data) and DAVID (all other data types) tools as described in the Sections from “Materials and Methods” to “Assessment of Biological Function*.”

**Table 5:** **Biological functions correlated with the percentage of CD4^+^CD28^−^ cells**.

	Biological function or pathway	Fold enrichment	*p*-Value^1^	Benjamini *q*-value^1^
CpG	Neuroactive ligand–receptor interaction	2.8	2.90E−05	3.50E–03
	Axon guidance	3.5	1.40E−04	8.50E–03
mRNA	None identified			
Protein	None identified			

	**Pathway**	***p*-Value^1^**	**# Genes**	**# miRNAs**

miRNA	ErbB signaling pathway	4.35E−34	44	9
	Regulation of actin cytoskeleton	2.38E−19	75	8
	Neurotrophin signaling pathway	8.49E−18	47	9
	Prostate cancer	2.68E−12	33	9
	MAPK signaling pathway	1.48E−11	78	9
	Focal adhesion	5.31E−11	62	9
	mTOR signaling pathway	4.31E−10	25	7
	Pathways in cancer	4.56E−10	100	9
	GnRH signaling pathway	1.27E−09	32	8
	Adherens junction	2.09E−09	29	8
	Chronic myeloid leukemia	1.03E−08	28	8
	Long-term potentiation	4.75E−08	25	8
	PI3K–Akt signaling pathway	6.37E−08	88	9
	Axon guidance	6.43E−08	43	8
	Acute myeloid leukemia	2.38E−07	21	8
	Endometrial cancer	3.04E−07	20	7
	Small cell lung cancer	7.34E−07	28	7
	Wnt signaling pathway	7.34E−07	44	9
	T cell receptor signaling pathway	2.81E−06	33	8
	Glioma	2.97E−06	25	9
	Renal cell carcinoma	3.25E−06	26	9
	Protein processing in endoplasmic reticulum	5.75E−06	50	8
	Pancreatic cancer	9.51E−06	24	8
	Insulin signaling pathway	1.09E−05	39	9
	Dilated cardiomyopathy	1.33E−05	28	7

*^1^*p*-values and *q*-values were determined by the DIANA (miRNA data) and DAVID (all other data types) tools as described in the Sections from “Materials and Methods” to “Assessment of Biological Function*.”

**Table 6:** **Biological functions correlated with percentage of CD8^+^CD28^−^ cells**.

	Biological function or pathway	Fold enrichment	*p*-Value^1^	Benjamini *q*-value^1^
CpG	Neuroactive ligand–receptor interaction	3.5	1.50E−08	1.80E−06
	Axon guidance	3.2	6.40E−04	3.90E−02
mRNA	Natural killer cell-mediated cytotoxicity	4.6	1.90E−05	0.0021
	Regulation of actin cytoskeleton	3.5	3.60E−05	0.0020
	Focal adhesion	3.5	6.80E−05	0.0026
	Graft-versus-host disease	8.5	1.40E−04	0.0040
	Antigen processing and presentation	4.5	0.0017	0.037
Protein	None identified			

	**Pathway**	***p*-Value^1^**	**# Genes**	**# miRNAs**

miRNA	PI3K–Akt signaling pathway	1.20E−21	117	7
	Prostate cancer	5.87E−20	40	6
	Ubiquitin-mediated proteolysis	6.24E−18	57	7
	Focal adhesion	9.26E−18	75	7
	Neurotrophin signaling pathway	4.11E−17	50	7
	ErbB signaling pathway	3.46E−15	38	6
	Glioma	6.79E−12	32	7
	Endometrial cancer	6.54E−11	24	6
	mTOR signaling pathway	1.79E−10	27	6
	Gap junction	1.96E−10	35	6
	Arrhythmogenic right ventricular cardiomyopathy	3.81E−10	33	7
	MAPK signaling pathway	4.44E−10	84	7
	Melanoma	6.11E−10	30	6
	Dilated cardiomyopathy	8.52E−10	35	7
	Aldosterone-regulated sodium reabsorption	8.84E−10	18	7
	Non-small cell lung cancer	1.02E−09	24	6
	Regulation of actin cytoskeleton	1.50E−09	72	6
	Chronic myeloid leukemia	3.97E−09	30	6
	Acute myeloid leukemia	3.97E−09	24	6
	Pancreatic cancer	8.48E−09	28	7
	Small cell lung cancer	1.44E−08	32	5
	Protein processing in endoplasmic reticulum	6.44E−08	57	7
	HIF-1 signaling pathway	8.77E−08	39	7
	Hypertrophic cardiomyopathy (HCM)	1.05E−07	31	7
	mRNA surveillance pathway	1.63E−07	32	6

*^1^*p*-values and *q*-values were determined by the DIANA (miRNA data) and DAVID (all other data types) tools as described in the Sections from “Materials and Methods” to “Assessment of Biological Function*.”

**Table 7:** **Biological functions correlated with CD4^+^/CD8^+^ T cell ratio**.

	Biological function or pathway	Fold enrichment	*p*-Value^1^	Benjamini *q*-value^1^
CpG	Axon guidance	3.4	5.40E−05	7.20E−03
mRNA	None identified			
Protein	None identified			

	**Pathway**	***p*-Value^1^**	**# Genes**	**# miRNAs**

miRNA	Neurotrophin signaling pathway	2.21E−10	27	4
	Long-term potentiation	1.55E−09	18	4
	Adherens junction	6.81E−09	18	3
	Regulation of actin cytoskeleton	1.68E−06	33	4
	Phosphatidylinositol signaling system	2.73E−06	19	4
	Colorectal cancer	1.62E−05	13	4
	Valine, leucine, and isoleucine biosynthesis	1.67E−05	2	2
	Endometrial cancer	1.70E−05	11	4
	Cholinergic synapse	2.17E−05	22	4
	Wnt signaling pathway	7.70E−05	25	4
	Axon guidance	7.70E−05	23	4
	Transcriptional misregulation in cancer	8.59E−05	25	4
	TGF-beta signaling pathway	0.00016	13	4
	Focal adhesion	0.00016	29	4
	Pantothenate and CoA biosynthesis	0.00020	6	2
	ErbB signaling pathway	0.00028	15	4
	Fc gamma R-mediated phagocytosis	0.00045	16	4
	Bacterial invasion of epithelial cells	0.00046	13	4
	Inositol phosphate metabolism	0.00056	13	4
	Dopaminergic synapse	0.00056	21	4
	Glioma	0.00056	13	4
	GnRH signaling pathway	0.0012	15	3
	Dilated cardiomyopathy	0.0014	15	3
	Endocytosis	0.0028	27	3
	Melanogenesis	0.0028	16	3

*^1^*p*-values and *q*-values were determined by the DIANA (miRNA data) and DAVID (all other data types) tools as described in the Sections “Materials and Methods” to “Assessment of Biological Function*.”

### Network Biology

**Figure 2:**
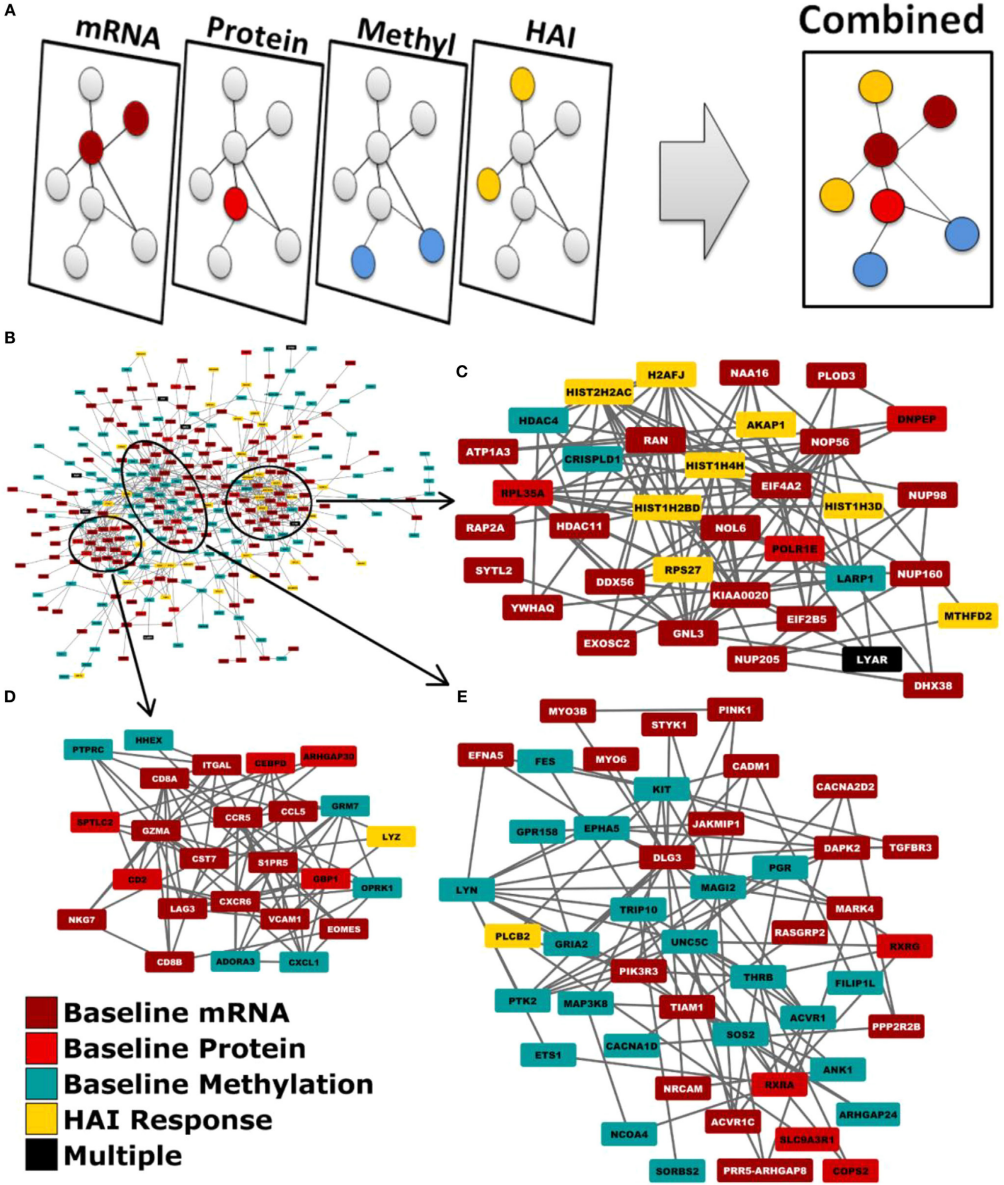
**Network biology integration of immunosenescence-related datasets**. **(A)** Each of our omics data types were mapped to genes and projected onto a common network. **(B)** This network diagram revealed functional links between the genes indicated by each data type. Three groups are evident within the network by the extent of their interconnectivity. **(C)** One group is highly concordant with M7.16 (*q* = 1.26xl0^−7^). **(D)** Another with the M3.6 cytotoxic T cell module (*q* = 6.31xl0^−5^). **(E)** The third is overlaps the canonical KEGG chemokine signaling (*q* = 3.98xl0^−5^).

We employed a network biology paradigm incorporating multiple network resources (see Materials and Methods) to integrate the results from the individual correlation analyses described above. A set of 138 canonical KEGG pathways describing distinct cellular functions remained after redundant and overlapping pathways were excluded. Genes were selected from our per-datatype analyses by *q*-value cut-offs such that no individual data type contributed more than 100 individual genes and did not dominate the integrated analysis. After extracting the subnetwork for genes associating with immunosenescence markers (see Figure 2), we identified multiple canonical cellular pathways as well as the previously described gene modules (see text footnote 1) (36). Genes within our subnetwork collectively overlapped six of these modules: M3.6 (Cytotoxic/NK cell function; *q* = 2.51 × 10^−8^) and the functionally undetermined modules: M9.18 (*q* = 1.26 × 10^−10^), M7.16 (*q* = 2.00 × 10^−6^), M9.7 (*q* = 6.31 × 10^−4^), M9.20 (*q* = 2.00 × 10^−3^), and M8.96 (*q* = 2.00 × 10^−3^). Within the subnetwork, three interconnected groups are evident. The first of these (Figure 2C) is highly concordant with M7.16 (*q* = 1.26 × 10^−7^). The second (Figure 2D) is highly associated with the M3.6 Cytotoxic module (*q* = 6.31 × 10^−5^) and also overlaps with the canonical chemokine signaling (*q* = 6.31 × 10^−3^), hematopoietic cell lineage (*q* = 7.94 × 10^−3^) and immunodeficiency (*q* = 1.58 × 10^−3^) KEGG pathways. The third (Figure 2E) overlaps the canonical chemokine signaling (*q* = 3.98 × 10^−5^) and axon guidance (*q* = 7.94 × 10^−4^) pathways.

## Discussion

### Immune Response Modeling and Immunosenescence

Participants in our cohort exhibited similar ranges of immunosenescence marker levels as observed in other recent studies (1–3, 5, 14, 51, 52). In our cohort, females had higher TREC levels and higher CD4/CD8 ratios than the male participants, suggesting that the females in our cohort may be less “immunosenescent” than males, as has been previously observed (53, 54). Our predictive modeling efforts indicated that immunosenescence explains only a small portion of inter-individual immune response variation in our cohort as only a few gene expression modules significantly associated with immune response also contained immunosenescence markers. One of these modules, Module M1.2 (36), is defined as an interferon module and contained primarily innate immune response-related genes/markers whose expression level was positively associated with HAI titer: *HERC5*, ubiquitin protein ligase 5, acts as a positive regulator of innate, interferon-induced, antiviral responses; the percent of CD8^+^CD28^−^ cells; *LY6E*, the lymphocyte antigen 6 complex that is activated by IFNα; as well as genes and markers negatively associated with HAI: chronological age; and *OAS3*, encoding the large OAS isoform that mediates antiviral activity through RNaseL.

Our findings also highlighted a cluster of histone genes from module M7.16, which does not have a defined immunologic function but can impact gene regulation. Our data suggest that the histone components of M7.16, along with multiple markers of immunosenescence, are involved in humoral immune responses to influenza vaccination. Despite the relative lack of an immunosenescence effect on humoral immunity in our cohort, the availability of additional, high-dimensional datasets (gene expression, miRNA expression, DNA methylation, and protein expression) provided an opportunity to examine, in depth, the impact of each immunosenescence marker on other biological functions.

### Age and Immunosenescence

Of the 70 CpG sites significantly correlated (*q* ≤ 0.15 and *p* ≤ 0.001) with age in our cohort, six of the top eight most significant (cg02228185, cg03032497, cg04875128, cg16983588, cg10501210, cg07955995) have been identified in previous aging studies (55–60). The CpG site with the most significant age-related correlation cg02228185 (in the *ASPA* gene) is one of three CpG sites identified as biomarkers of human aging (61). The other two CpG sites identified in that study are cg25809905 and cg17861230, which missed our FDR threshold (*p* = 1.6 × 10^−4^, *q* = 0.16 and *p* = 1.8 × 10^−2^, *q* > 0.2, respectively).

Many of the top age-associated CpGs are within genes controlling immune function (Table S1 in Supplementary Material). For example, *KLF14* has one of the strongest signals (ρ = 0.39, *p* = 4.77 × 10^−7^) and is a transcription factor that regulates TGFβ signaling by modulating TGFβ receptor expression, suggesting that expression of *KLF4* may wane with age. A CpG in the promoter of *HLA-DPB1* is negatively correlated with age, potentially indicating greater expression in older adults. *TSPAN33* wanes with age and is known to play a role in red blood cell differentiation. These age-associated changes in methylation may help determine the landscape of potential immune response across our study cohort.

Age-correlated CpG sites were in general concordance with age-related methylation profiles in monocytes, as previously reported by Reynolds et al. (51), which indicates that many of these previously identified epigenetic trends in monocytes can also be identified within a heterogeneous cell population (PBMCs), albeit at a diminished level as would be expected due to the presence of other cell types that dilute the signal. The novel age-methylation associations discovered in this report are likely driven by specific cell subsets not examined in previous studies.

Regarding individual genes with expression levels associated with aging, Nakamura et al. identified 16 transcripts that were highly correlated with age (62). Vo et al. studied transcriptomic markers of aging in PBMCs and identified a separate set of 16 genes whose expression levels correlated with age (63). While we noted similar trends with these genes, none of them met our false discovery threshold. The differences in experimental approaches (RNA isolation from whole blood instead of PBMCs), age ranges (23–77 versus 50–74) make it difficult to make direct comparisons between these studies and ours. Interestingly, despite these differences, six of the 16 age-related genes in the Nakamura study (*CTSD, TNFRSF1B, CTSS, GAA, CTSH*, and *CD28)* and two of the 16 age-related genes from the Vo study (*MXRA8* and *DDB2*) were correlated with TERT levels. This degree of overlap suggests that age and TERT activity are related and that both should be considered when characterizing immunosenescence, as well as when exploring the underlying mechanisms.

We identified two proteins whose expression levels were positively correlated with age. One of the proteins is the EGF-Response Factor 2 (encoded by *ZFP36L2*), a negative regulator of erythroid differentiation. Dysregulation of EGF activity has also been described during cellular senescence of long-term cultured T cell clones (64). Elevated levels during aging may affect the body’s ability to replenish lymphocyte populations, as well as the ability of aging cells to function properly. The second protein is the polypeptide E component of RNA polymerase I that mediates formation of the initiation complex at the promoter.

With respect to age and miRNA expression, Gombar et al. identified miRNA-363* expression decreasing with age, but remained elevated in centenarians (65). In our cohort, miRNA 363-3p exhibited a negative correlation with the percentage of CD4^+^CD28^−^ cells (*r* = −0.39, *p* = 0.004, *q* = 0.06), which supports the assertion that miR-363 may be associated with longevity.

Another recent study examining genome-wide miRNA signatures identified and validated eight miRNAs associated with aging (66). Despite differences in study design, biospecimen collection, miRNA sequencing, and analysis, three of the eight miRNAs were also associated with markers of immunosenescence in our subjects: miR-320a (*r* = −0.44, *p* = 0.0010, *q* = 0.036) was associated with percentage of CD4^+^CD28^−^ cells; and both miR-320b (*r* = 0.40, *p* = 0.0037, *q* = 0.12) and miR-320d (*r* = 0.43, *p* = 0.0016, *q* = 0.095) were associated with the CD4^+^/CD8^+^ ratio. miR-320 controls the inhibition of cell division and regulates glycolysis (67) and may also control signaling through the Wnt and insulin PI3K pathways (68, 69). Dysregulation of miRNA expression during aging may impair critical signaling pathways necessary for immune function.

### Telomerase, TREC, and Immunosenescence

In our cohort, we identified a number of genes, epigenetic regulatory elements, and protein markers correlated with telomerase or TREC expression. Interestingly, miRNAs associated with TERT expression controlled a number of important pathways associated with aging and with immune function, including the mTOR, MAPK, p53, and PI3K signaling pathways. TREC levels were associated with miRNAs controlling a similar array of signaling pathways (MAPK, Wnt, ErbB, PI3K-Akt, calcium), as well as the B cell receptor signaling pathway, perhaps reflecting the role of these pathways in the activation and differentiation of naïve lymphocytes. Our data suggest that miRNA-mediated control of these pathways may contribute to both aging and immunosenescence.

### CD4^+^/CD8^+^ T Cells Lacking Expression of CD28 and Immunosenescence

CD28 expression has been found to correlate with replicative ability and increases in the CD28^−^ compartment of both CD4^+^ and CD8^+^ T cells is an indicator of immunosenescence (70, 71). It has been proposed that CD28 downregulation occurs in response to chronic immune stimulation in older individuals (72). Our results demonstrated a remarkable amount of overlap in both the individual variables (CpG sites, miRNAs, mRNAs) and the pathways/biological functions that correlated with either the percent of CD4^+^ or the percent of CD8^+^ T cells lacking CD28.

### CD4^+^/CD8^+^ T Cell Ratio and Immunosenescence

An individual’s CD4/CD8 ratio has been used as a predictor of mortality, a component of an immune risk phenotype, and has been used to monitor HIV progression (49, 73, 74). A higher ratio in females has been previously reported (75), and there is an indication that the ratio is controlled by multiple genetic factors. Our data indicate that individuals with a high CD4/CD8 ratio had gene expression profiles enriched in pathways directly contributing to lymphocyte function (i.e., NK cell function, TCR signaling pathway, leukocyte migration, antigen processing), although further work will be necessary in order to determine if differential gene expression is the result of or cause of the differences in CD4/CD8 ratio.

### Network Biology and Integrated Analysis of Immunosenescence

Individually, each data type (mRNA expression, miRNA expression, DNA methylation, protein expression) highlighted several biological activities correlated with markers of immunosenescence. Collectively, our integrated network-based analysis provided further insights into immunosenescence and indicated that alterations in signaling pathways span multiple complex biological spaces. A more complete picture emerges (Figure 2) once the data are analyzed together. Importantly, changes in activity may not result from changes in gene expression or protein abundance, but can be due to post-translational modification, changes in localization, etc. (76, 77). This integrated analysis highlighted genesets/pathways known to be dysregulated during immunosenescence: chemokine signaling, immunodeficiency, cytotoxic T cell activity, and hematopoietic cell lineage. Of note, we also identified five gene modules with previously undetermined function (M7.16, M8.96, M9.7, M9.18, and M9.20) that are associated with markers of immunosenescence. Further study is needed to elucidate the biological mechanisms regulated by these genesets, but their role in immunosenescence makes them interesting candidates for their potential to modulate immune responses.

Within the subnetwork, three groups of genes are evident by their mutual associations within our integrated network resource. Each of these groups represent the genes corresponding to canonical pathways and network modules represented within the subnetwork (Figure 2). The first group encompasses nearly all of the genes contributing to enrichment of M7.16. The second group encompasses nearly all M3.6 genes and the third, the axon guidance canonical pathway. While each group contained genes from multiple modules or pathways, one module/pathway always had greater representation than the others and was, therefore, identified as the group’s primary function. Externally defined canonical pathways and modules are believed to represent real cellular functions due to established biochemical and genetic assays demonstrating the encoded relationships between genes, and their recurrent activity changes in multiple studies. Our network biology approach identified an interconnected set of modules and pathways that were associated immunosenescence markers and are co-regulated by multiple biologic mechanisms, giving us enhanced confidence in our findings. Our results suggest that these diverse biological activities may be controlled by shared regulatory features that are dysregulated during immunosenescence. This information may provide a starting point to adapt future vaccination efforts to aging and immunosenescent populations.

### Strengths and Limitations of the Study

Our study has several notable strengths: (1) the use of datasets derived from multiple biological spaces (mRNA, miRNA, protein, cell subsets, functional immune outcomes) for the same samples; (2) the comprehensive nature of each dataset; (3) the range of immunosenescence markers studied; and (4) the network biology approach facilitating the integrated analysis and interpretation of biological functions associated with immunosenescence markers. One limitation of our study is that our cohort only spans ages 50–74 years, which may not reflect the full spectrum of declining immune function. Our subjects were also selected based on good general health and stable medication use. It is possible that this resulted in a population that is more immunocompetent than the general population. The immunosenescence effects characterized by the markers we have measured may have already achieved their major impact on immune function by the minimum age sampled, and a direct comparison with a young population is not available. Despite the limited age range of our cohort, we were able to successfully identify multiple factors across different biological spaces (e.g., genes, miRNAs, CpG sites, proteins, lymphocyte subsets) associated with common immunosenescence markers. Another limitation of studies involving high-dimensional data is the possibility of false positives, which we control for through the use of both *p*-value and *q*-value thresholds. Our study utilized PBMCs rather than a specific cell subset (NK cells, plasmacytoid DCs, effector memory CD8^+^ T cells) and the results represent the collective response of multiple cell types. While this approach allows for ease of biospecimen collection, reduces *ex vivo* manipulation, and better approximates the *in vivo* situation, it does have some disadvantages, including the presence of multiple signals due to cell type-specific responses increasing the “noise” present in the data, and the inability to track identified transcriptomic signatures back to a defined cell population.

Our cohort consisted of older individuals from a population with high vaccination rates. It is reasonable to assume that our cohort all received the monovalent, pandemic influenza A/H1N1 vaccine in 2009 and that the vaccination studied in this report is their second immunization against pandemic influenza A/H1N1. Due to the age range of this cohort, it is likely that they have had multiple prior exposures to various influenza strains. One possible explanation for the weak associations between markers of immunosenescence and humoral immunity in this population is that repeat immunization may partially overcome the effects of immunosenescence. For example, booster vaccines may increase the number of antigen-specific T cells, known to correlate with protection in older adults (78).

## Conclusion

Our findings provide further evidence that immunosenescence is a complex phenomenon involving multiple aspects of both innate and adaptive immunity (79, 80). We believe that a systems level vaccinomics approach will be necessary in order to capture not just the individual defects of specific cell subsets, but also the altered interactions, networks, and signaling pathways that are dysregulated during the aging process (14, 32, 61, 62, 81–88). Our work has started this process by linking immunosenescence markers with transcriptomic and epigenomic factors and their associated functional pathways. The identified associations were found in pathways controlling cell proliferation, oncogenesis, and hormone regulation, as well as the metabolic activities likely to be necessary for activation, proliferation, and differentiation of lymphocyte subsets. These pathways also undergo significant changes during the aging process and the phenotypic changes that we collectively categorize as “immunosenescence” may be symptoms of a more fundamental breakdown in cellular function.

An enhanced understanding of immunosenescence has profound implications for vaccine use in older populations. Vaccine efficacy is severely compromised in the elderly. This is due, in large part, to immunosenescence. The ability to define the key processes that are disrupted during immunosenescence may also allow us to develop new adjuvants, delivery systems, and formulations that overcome those deficits and stimulate robust, protective immune responses. The high-dose formulation of influenza vaccination is one example of an attempt to overcome reduced immune function through increased antigen dose. Alternative approaches may rely on the stimulation of specific immune responses; for example, the newly licensed MF59-adjuvanted influenza vaccine demonstrated to have higher immunogenicity and result in longer-lived antibody responses following vaccination (89–91). Additional examples include the use of GLA-SE (a TLR4 agonist) leading to increased production of TNFα, IL-6, and IL-12, as well as a Th1 shift in response to influenza challenge (92); or the use of Ov-ASP-1 (a helminth-derived protein adjuvant that stimulates both Th1 and Th2 responses with resulting increases in influenza-specific antibody titer in both young and old mice) (93). This knowledge may also provide useful biomarkers for the testing and evaluation of vaccine candidates. T cell responses have been shown to be better correlates of protection in the elderly than antibody titer (78). This knowledge, combined with results demonstrating the presence of T cell epitopes in additional influenza proteins (94, 95), may lead to vaccines with an altered composition designed to be maximally immunogenic in the elderly (96). Our own results indicate that, in addition to alterations in immune pathways, metabolic activity and other housekeeping processes are also dysregulated. With this in mind, treatments (e.g., vitamins or nutritional supplements) that correct imbalanced or dysregulated metabolic or other physiologic processes may be administered to individuals prior to receipt of current vaccine products. The next important step for the field will be to begin systematically evaluating the identified immunologic and immunosenescence-related molecular signatures as biomarkers, and for targeted manipulation in order to shape immune response outcomes and elicit protective immunity in vulnerable populations.

## Author Contributions

RK participated in the conception and design of the study, carried out the immune assays, assisted in data interpretation and analysis, and drafted the manuscript. IO participated in the design of the study, carried out immune assays, and assisted in drafting the manuscript. IH participated in the design of the study, carried out immune assays, and assisted in drafting of the manuscript. AO participated in the design of the study, planned and directed the statistical analyses, and helped draft the manuscript. MZ carried out bioinformatic analyses and helped draft the manuscript. DG participated in the design of the study, carried out statistical analyses, and helped draft the manuscript. GP conceived of the study, participated in its design, helped to interpret the data, and participated in writing/revising the manuscript. All authors read, critiqued, and approved the final manuscript. All authors also agree to be accountable for all aspects of the work.

## Conflict of Interest Statement

Dr. RK has grant funding from Merck Research Laboratories to study waning immunity after mumps vaccination. Dr. GP is the chair of a Safety Evaluation Committee for novel investigational vaccine trials being conducted by Merck Research Laboratories. Dr. GP offers consultative advice on vaccine development to Merck & Co. Inc., CSL Biotherapies, Avianax, Dynavax, Novartis Vaccines and Therapeutics, Emergent Biosolutions, Adjuvance, and Microdermis. Drs. GP and IO hold two patents related to vaccinia and measles peptide research. These activities have been reviewed by the Mayo Clinic Conflict of Interest Review Board and are conducted in compliance with Mayo Clinic Conflict of Interest policies. This research has been reviewed by the Mayo Clinic Conflict of Interest Review Board and was conducted in compliance with Mayo Clinic Conflict of Interest policies. The remaining authors declare that the research was conducted in the absence of any commercial or financial relationships that could be construed as a potential conflict of interest.

## Footnotes

^1^http://www.biir.net/public_wikis/module_annotation/G2_Trial_8_Modules.

^2^http://diana.imis.athena-innovation.gr/DianaTools/.

^3^http://david.abcc.ncifcrf.gov/home.jsp.
