# Supplementary material for: Immunosenescence-Related Transcriptomic and Immunologic Changes in Older Individuals Following Influenza Vaccination
Source: Front Immunol. 2016 Nov 2;7:450. doi: 10.3389/fimmu.2016.00450 (PMC5089977; doi:10.3389/fimmu.2016.00450)
Supplement: Supplementary file 3 [file Table_3.DOCX]

| **Supplemental Table 3. miRNA-Controlled Pathways Correlated with Immunosenescence** | | | | | | | |
| --- | --- | --- | --- | --- | --- | --- | --- |
| **Age** | | | | | | | |
| **Pathway** | **p**  **value** | **#**  **genes** | **#**  **miRNAs** | **Pathway** | **p**  **value** | **#**  **genes** | **#**  **miRNAs** |
| No miRNAs with significant correlations |  |  |  | No miRNAs with significant correlations |  |  |  |
| **TREC** | | | | | | | |
| **Pathway** | **p**  **value** | **#**  **genes** | **#**  **miRNAs** | **Pathway** | **p**  **value** | **#**  **genes** | **#**  **miRNAs** |
| MAPK signaling pathway | 6.22E-10 | 40 | 5 | Hypertrophic cardiomyopathy (HCM) | 0.016 | 11 | 5 |
| Neurotrophin signaling pathway | 2.02E-06 | 20 | 5 | p53 signaling pathway | 0.024 | 9 | 4 |
| Ubiquitin mediated proteolysis | 6.49E-05 | 21 | 5 | Hepatitis B | 0.038 | 16 | 4 |
| D-Glutamine and D-glutamate metabolism | 0.00035 | 2 | 2 | Drug metabolism - cytochrome P450 | 0.039 | 3 | 2 |
| GnRH signaling pathway | 0.00038 | 14 | 5 | Histidine metabolism | 0.039 | 5 | 3 |
| Insulin signaling pathway | 0.0020 | 18 | 5 | Amyotrophic lateral sclerosis (ALS) | 0.039 | 8 | 3 |
| RNA transport | 0.0020 | 18 | 6 | B cell receptor signaling pathway | 0.039 | 10 | 4 |
| Long-term potentiation | 0.0025 | 11 | 5 | Acute myeloid leukemia | 0.039 | 8 | 4 |
| PI3K-Akt signaling pathway | 0.0047 | 35 | 5 | Valine, leucine and isoleucine biosynthesis | 0.042 | 1 | 1 |
| Calcium signaling pathway | 0.0054 | 21 | 5 | Gap junction | 0.042 | 12 | 4 |
| Small cell lung cancer | 0.0069 | 12 | 4 | Arrhythmogenic right ventricular cardiomyopathy | 0.043 | 12 | 5 |
| Dilated cardiomyopathy | 0.011 | 12 | 5 | Chemokine signaling pathway | 0.044 | 19 | 5 |
| T cell receptor signaling pathway | 0.012 | 14 | 4 |  |  |  |  |
| **TERT** | | | | | | | |
| **Pathway** | **p**  **value** | **#**  **genes** | **#**  **miRNAs** | **Pathway** | **p**  **value** | **#**  **genes** | **#**  **miRNAs** |
| Endometrial cancer | 0.00064 | 4 | 1 | Small cell lung cancer | 0.012 | 4 | 1 |
| Regulation of actin cytoskeleton | 0.00070 | 8 | 1 | GnRH signaling pathway | 0.017 | 4 | 1 |
| p53 signaling pathway | 0.0016 | 4 | 1 | Prostate cancer | 0.017 | 4 | 1 |
| Glioma | 0.0016 | 4 | 1 | Dorso-ventral axis formation | 0.022 | 2 | 1 |
| ErbB signaling pathway | 0.0032 | 4 | 1 | Thyroid cancer | 0.022 | 2 | 1 |
| D-Glutamine and D-glutamate metabolism | 0.0053 | 1 | 1 | PI3K-Akt signaling pathway | 0.031 | 8 | 1 |
| Acute myeloid leukemia | 0.011 | 3 | 1 | mTOR signaling pathway | 0.037 | 3 | 1 |
| Neurotrophin signaling pathway | 0.012 | 5 | 1 | MAPK signaling pathway | 0.043 | 7 | 1 |
| **% CD28^-^ CD4 T cells** | | | | | | | |
| **Pathway** | **p**  **value** | **#**  **genes** | **#**  **miRNAs** | **Pathway** | **p**  **value** | **#**  **genes** | **#**  **miRNAs** |
| ErbB signaling pathway | 7.43E-39 | 48 | 10 | Protein processing in endoplasmic reticulum | 2.04E-05 | 54 | 9 |
| Prostate cancer | 1.13E-25 | 42 | 10 | VEGF signaling pathway | 2.77E-05 | 23 | 7 |
| Regulation of actin cytoskeleton | 3.49E-23 | 84 | 9 | Hepatitis B | 3.09E-05 | 46 | 9 |
| Neurotrophin signaling pathway | 5.94E-23 | 53 | 10 | B cell receptor signaling pathway | 4.48E-05 | 25 | 8 |
| Focal adhesion | 4.50E-19 | 75 | 10 | TGF-beta signaling pathway | 7.53E-05 | 28 | 9 |
| Endometrial cancer | 7.68E-16 | 25 | 8 | Dopaminergic synapse | 9.10E-05 | 39 | 9 |
| Adherens junction | 3.41E-15 | 34 | 9 | Nicotine addiction | 9.83E-05 | 17 | 8 |
| Renal cell carcinoma | 8.58E-15 | 34 | 10 | Bladder cancer | 0.00011 | 16 | 9 |
| mTOR signaling pathway | 8.83E-15 | 29 | 8 | p53 signaling pathway | 0.00016 | 23 | 7 |
| PI3K-Akt signaling pathway | 2.25E-14 | 107 | 10 | RNA degradation | 0.00016 | 24 | 9 |
| Axon guidance | 2.30E-14 | 51 | 9 | RNA transport | 0.00021 | 46 | 9 |
| Pathways in cancer | 2.45E-14 | 114 | 10 | Viral carcinogenesis | 0.00048 | 60 | 9 |
| MAPK signaling pathway | 1.55E-13 | 87 | 10 | Fc gamma R-mediated phagocytosis | 0.00049 | 29 | 6 |
| Chronic myeloid leukemia | 2.92E-13 | 32 | 9 | Circadian rhythm | 0.0014 | 12 | 6 |
| Long-term potentiation | 5.21E-10 | 28 | 9 | Chemokine signaling pathway | 0.0016 | 51 | 10 |
| Melanoma | 7.50E-10 | 29 | 9 | Valine, leucine and isoleucine degradation | 0.0022 | 14 | 6 |
| Acute myeloid leukemia | 1.53E-09 | 24 | 9 | Osteoclast differentiation | 0.0022 | 38 | 9 |
| Insulin signaling pathway | 1.76E-08 | 46 | 10 | Glutamatergic synapse | 0.0023 | 35 | 8 |
| GnRH signaling pathway | 1.92E-08 | 33 | 9 | Valine, leucine and isoleucine biosynthesis | 0.0034 | 2 | 3 |
| Glioma | 2.96E-08 | 29 | 10 | Cocaine addiction | 0.0043 | 19 | 8 |
| Dilated cardiomyopathy | 1.26E-07 | 32 | 8 | Gastric acid secretion | 0.0043 | 23 | 8 |
| Pancreatic cancer | 1.27E-07 | 27 | 9 | Adipocytokine signaling pathway | 0.0043 | 21 | 10 |
| Hypertrophic cardiomyopathy (HCM) | 1.27E-07 | 30 | 9 | HIF-1 signaling pathway | 0.0046 | 32 | 10 |
| T cell receptor signaling pathway | 1.69E-07 | 37 | 9 | Type II diabetes mellitus | 0.0057 | 15 | 7 |
| Wnt signaling pathway | 2.46E-07 | 49 | 10 | Progesterone-mediated oocyte maturation | 0.0057 | 25 | 8 |
| Non-small cell lung cancer | 5.46E-07 | 22 | 9 | Lysine degradation | 0.0068 | 17 | 8 |
| Shigellosis | 6.23E-07 | 24 | 8 | Gap junction | 0.0068 | 28 | 8 |
| Small cell lung cancer | 6.23E-07 | 30 | 8 | Amoebiasis | 0.0085 | 30 | 9 |
| Bacterial invasion of epithelial cells | 6.85E-07 | 27 | 7 | Cholinergic synapse | 0.0085 | 35 | 8 |
| Thyroid cancer | 1.47E-06 | 13 | 6 | Inositol phosphate metabolism | 0.011 | 20 | 5 |
| Aldosterone-regulated sodium reabsorption | 1.47E-06 | 16 | 7 | Basal cell carcinoma | 0.012 | 17 | 8 |
| Dorso-ventral axis formation | 2.40E-06 | 11 | 6 | Long-term depression | 0.015 | 21 | 7 |
| Melanogenesis | 2.84E-06 | 34 | 10 | Alanine, aspartate and glutamate metabolism | 0.016 | 11 | 6 |
| Ubiquitin mediated proteolysis | 5.24E-06 | 44 | 9 | Measles | 0.018 | 39 | 8 |
| Arrhythmogenic right ventricular cardiomyopathy (ARVC) | 1.06E-05 | 30 | 8 | Biotin metabolism | 0.024 | 1 | 2 |
| Fc epsilon RI signaling pathway | 1.34E-05 | 25 | 8 | Retrograde endocannabinoid signaling | 0.024 | 32 | 9 |
| Colorectal cancer | 1.34E-05 | 23 | 8 | Protein digestion and absorption | 0.032 | 24 | 9 |
| Transcriptional misregulation in cancer | 1.92E-05 | 58 | 9 | HTLV-I infection | 0.042 | 65 | 10 |
| mRNA surveillance pathway | 2.03E-05 | 30 | 9 |  |  |  |  |
| **% CD28^-^ CD8 T cells** | | | | | | | |
| **Pathway** | **p**  **value** | **#**  **genes** | **#**  **miRNAs** | **Pathway** | **p**  **value** | **#**  **genes** | **#**  **miRNAs** |
| PI3K-Akt signaling pathway | 1.20E-21 | 117 | 7 | Dopaminergic synapse | 7.58E-05 | 40 | 7 |
| Prostate cancer | 5.87E-20 | 40 | 6 | Phosphatidylinositol signaling system | 9.42E-05 | 31 | 6 |
| Ubiquitin mediated proteolysis | 6.24E-18 | 57 | 7 | p53 signaling pathway | 0.00015 | 23 | 5 |
| Focal adhesion | 9.26E-18 | 75 | 7 | GnRH signaling pathway | 0.00017 | 29 | 7 |
| Neurotrophin signaling pathway | 4.11E-17 | 50 | 7 | TGF-beta signaling pathway | 0.00020 | 30 | 5 |
| ErbB signaling pathway | 3.46E-15 | 38 | 6 | Nicotine addiction | 0.00026 | 17 | 6 |
| Glioma | 6.79E-12 | 32 | 7 | B cell receptor signaling pathway | 0.00027 | 25 | 6 |
| Endometrial cancer | 6.54E-11 | 24 | 6 | Pancreatic secretion | 0.00045 | 30 | 6 |
| mTOR signaling pathway | 1.79E-10 | 27 | 6 | Cholinergic synapse | 0.00053 | 38 | 6 |
| Gap junction | 1.96E-10 | 35 | 6 | Lysine degradation | 0.00061 | 18 | 6 |
| Arrhythmogenic right ventricular cardiomyopathy | 3.81E-10 | 33 | 7 | Renal cell carcinoma | 0.00081 | 26 | 7 |
| MAPK signaling pathway | 4.44E-10 | 84 | 7 | Axon guidance | 0.00089 | 42 | 6 |
| Melanoma | 6.11E-10 | 30 | 6 | T cell receptor signaling pathway | 0.00089 | 33 | 6 |
| Dilated cardiomyopathy | 8.52E-10 | 35 | 7 | Wnt signaling pathway | 0.0018 | 46 | 6 |
| Aldosterone-regulated sodium reabsorption | 8.84E-10 | 18 | 7 | Bacterial invasion of epithelial cells | 0.0022 | 25 | 6 |
| Non-small cell lung cancer | 1.02E-09 | 24 | 6 | Salivary secretion | 0.0022 | 27 | 7 |
| Regulation of actin cytoskeleton | 1.50E-09 | 72 | 6 | VEGF signaling pathway | 0.0025 | 21 | 6 |
| Chronic myeloid leukemia | 3.97E-09 | 30 | 6 | Terpenoid backbone biosynthesis | 0.0027 | 8 | 4 |
| Acute myeloid leukemia | 3.97E-09 | 24 | 6 | Progesterone-mediated oocyte maturation | 0.0030 | 26 | 6 |
| Pancreatic cancer | 8.48E-09 | 28 | 7 | Carbohydrate digestion and absorption | 0.0036 | 14 | 6 |
| Small cell lung cancer | 1.44E-08 | 32 | 5 | Adipocytokine signaling pathway | 0.0040 | 22 | 7 |
| Protein processing in endoplasmic reticulum | 6.44E-08 | 57 | 7 | Valine, leucine and isoleucine biosynthesis | 0.0041 | 2 | 5 |
| HIF-1 signaling pathway | 8.77E-08 | 39 | 7 | Type II diabetes mellitus | 0.0041 | 16 | 6 |
| Hypertrophic cardiomyopathy (HCM) | 1.05E-07 | 31 | 7 | Dorso-ventral axis formation | 0.0045 | 9 | 5 |
| mRNA surveillance pathway | 1.63E-07 | 32 | 6 | Inositol phosphate metabolism | 0.0053 | 22 | 5 |
| Calcium signaling pathway | 1.82E-07 | 58 | 7 | Retrograde endocannabinoid signaling | 0.0053 | 35 | 6 |
| Endocytosis | 3.27E-07 | 65 | 6 | Viral carcinogenesis | 0.0053 | 58 | 7 |
| Transcriptional misregulation in cancer | 1.30E-06 | 60 | 7 | Endocrine and other factor-regulated calcium reabsorption | 0.0074 | 19 | 6 |
| Pathways in cancer | 1.31E-06 | 103 | 7 | RNA transport | 0.0077 | 43 | 7 |
| Adherens junction | 4.14E-06 | 29 | 6 | Folate biosynthesis | 0.016 | 5 | 2 |
| Amoebiasis | 4.14E-06 | 36 | 7 | Bile secretion | 0.019 | 22 | 7 |
| Long-term potentiation | 5.01E-06 | 25 | 7 | Fanconi anemia pathway | 0.020 | 17 | 5 |
| Insulin signaling pathway | 1.12E-05 | 43 | 7 | Fc epsilon RI signaling pathway | 0.020 | 21 | 6 |
| Bladder cancer | 1.56E-05 | 17 | 7 | RNA degradation | 0.027 | 21 | 7 |
| Chemokine signaling pathway | 3.68E-05 | 55 | 7 | Biotin metabolism | 0.031 | 1 | 2 |
| Gastric acid secretion | 7.13E-05 | 26 | 7 | Melanogenesis | 0.043 | 28 | 7 |
| **CD4/CD8 T cell ratio** | | | | | | | |
| **Pathway** | **p**  **value** | **#**  **genes** | **#**  **miRNAs** | **Pathway** | **p**  **value** | **#**  **genes** | **#**  **miRNAs** |
| Neurotrophin signaling pathway | 2.21E-10 | 27 | 4 | Melanogenesis | 0.0028 | 16 | 3 |
| Long-term potentiation | 1.55E-09 | 18 | 4 | Calcium signaling pathway | 0.0032 | 24 | 3 |
| Adherens junction | 6.81E-09 | 18 | 3 | mTOR signaling pathway | 0.0046 | 11 | 3 |
| Regulation of actin cytoskeleton | 1.68E-06 | 33 | 4 | Thyroid cancer | 0.0063 | 6 | 3 |
| Phosphatidylinositol signaling system | 2.73E-06 | 19 | 4 | PI3K-Akt signaling pathway | 0.0086 | 37 | 4 |
| Colorectal cancer | 1.62E-05 | 13 | 4 | Hypertrophic cardiomyopathy (HCM) | 0.0086 | 13 | 3 |
| Valine, leucine and isoleucine biosynthesis | 1.67E-05 | 2 | 2 | Gastric acid secretion | 0.0086 | 12 | 4 |
| Endometrial cancer | 1.70E-05 | 11 | 4 | Salivary secretion | 0.014 | 13 | 4 |
| Cholinergic synapse | 2.17E-05 | 22 | 4 | Pathogenic Escherichia coli infection | 0.015 | 9 | 4 |
| Wnt signaling pathway | 7.70E-05 | 25 | 4 | Notch signaling pathway | 0.016 | 8 | 3 |
| Axon guidance | 7.70E-05 | 23 | 4 | Prostate cancer | 0.018 | 13 | 4 |
| Transcriptional misregulation in cancer | 8.59E-05 | 25 | 4 | Dorso-ventral axis formation | 0.018 | 5 | 2 |
| TGF-beta signaling pathway | 0.00016 | 13 | 4 | Lysine degradation | 0.019 | 8 | 4 |
| Focal adhesion | 0.00016 | 29 | 4 | Shigellosis | 0.019 | 9 | 4 |
| Pantothenate and CoA biosynthesis | 0.00020 | 6 | 2 | Chagas disease (American trypanosomiasis) | 0.019 | 15 | 4 |
| ErbB signaling pathway | 0.00028 | 15 | 4 | RNA degradation | 0.023 | 11 | 2 |
| Fc gamma R-mediated phagocytosis | 0.00045 | 16 | 4 | Amyotrophic lateral sclerosis (ALS) | 0.023 | 9 | 4 |
| Bacterial invasion of epithelial cells | 0.00046 | 13 | 4 | Small cell lung cancer | 0.026 | 12 | 4 |
| Inositol phosphate metabolism | 0.00056 | 13 | 4 | Pancreatic cancer | 0.030 | 10 | 4 |
| Dopaminergic synapse | 0.00056 | 21 | 4 | MAPK signaling pathway | 0.031 | 30 | 4 |
| Glioma | 0.00056 | 13 | 4 | B cell receptor signaling pathway | 0.035 | 11 | 4 |
| GnRH signaling pathway | 0.0012 | 15 | 3 | Protein processing in endoplasmic reticulum | 0.036 | 21 | 3 |
| Dilated cardiomyopathy | 0.0014 | 15 | 3 | Ubiquitin mediated proteolysis | 0.049 | 17 | 3 |
| Endocytosis | 0.0028 | 27 | 3 |  |  |  |  |
