# Supplementary material for: Immunosenescence-Related Transcriptomic and Immunologic Changes in Older Individuals Following Influenza Vaccination
Source: Front Immunol. 2016 Nov 2;7:450. doi: 10.3389/fimmu.2016.00450 (PMC5089977; doi:10.3389/fimmu.2016.00450)
Supplement: Supplementary file 5 [file Table_5.DOCX]

| **Supplemental Table 5. Protein Expression Correlated with Immunosenescence** | | | | | | | | |
| --- | --- | --- | --- | --- | --- | --- | --- | --- |
| **Age** | | | **TREC** | | | **% CD28^-^**  **CD4 T cells** | | |
| **Protein** | **Corr.** | **p-value** | **Protein** | **Corr.** | **p-value** | **Protein** | **Corr.** | **p-value** |
| ZFP36L2 | 0.68 | 2.03E-05 | DNPEP | 0.56 | 2.69E-05 | GPR56 | 0.64 | 5.28E-05 |
| POLR1E | 0.58 | 3.96E-05 | ALDH5A1 | 0.54 | 4.41E-05 | GZMH | 0.64 | 6.93E-07 |
|  |  |  | SATB1 | 0.53 | 7.92E-05 | CD2 | 0.61 | 2.36E-06 |
|  |  |  | C3orf37 | 0.52 | 0.00016 | IDS | 0.55 | 3.37E-05 |
|  |  |  | COPS2 | 0.51 | 0.00014 | PYHIN1 | 0.55 | 3.57E-05 |
|  |  |  | RPL35A | 0.49 | 0.00026 | AGA | 0.53 | 7.31E-05 |
|  |  |  | RXRA;RXRG | -0.50 | 0.00031 | PPP1R18 | 0.53 | 8.34E-05 |
|  |  |  | CEBPD | -0.50 | 0.00025 | SLC9A3R1 | 0.52 | 0.00011 |
|  |  |  | APOBR | -0.51 | 0.00018 | LYAR | 0.51 | 0.00013 |
|  |  |  | SPTLC2 | -0.52 | 0.00010 | RNF213 | 0.51 | 0.00018 |
|  |  |  | AGPS | -0.52 | 9.76E-05 | APOL2 | 0.51 | 0.00018 |
|  |  |  | ATL3 | -0.55 | 3.31E-05 | GBP1 | 0.49 | 0.00030 |
|  |  |  | AHR | -0.63 | 6.41E-05 | WDR55 | 0.48 | 0.00036 |
|  |  |  |  |  |  | UPP1 | 0.48 | 0.00037 |
|  |  |  |  |  |  | ARHGAP30 | 0.48 | 0.00039 |
|  |  |  |  |  |  | MROH1 | -0.50 | 0.00029 |
