# Supplementary material for: Immunosenescence-Related Transcriptomic and Immunologic Changes in Older Individuals Following Influenza Vaccination
Source: Front Immunol. 2016 Nov 2;7:450. doi: 10.3389/fimmu.2016.00450 (PMC5089977; doi:10.3389/fimmu.2016.00450)
Supplement: Supplementary file 6 [file Table_6.docx]

| **Supplemental Table 7. Cellular Immunophenotypes Correlated with Immunosenescence Markers** | | | | | |
| --- | --- | --- | --- | --- | --- |
| **Age** | | | | | |
| **Phenotype** | **Correlation** | **p-value** | **Phenotype** | **Correlation** | **p-value** |
|  |  |  |  |  |  |
| **Telomerase** | | | | | |
| **Phenotype** | **Correlation** | **p-value** | **Phenotype** | **Correlation** | **p-value** |
|  |  |  | % Non-classical monocytes | -0.28 | 0.0003 |
| **TREC** | | | | | |
| **Phenotype** | **Correlation** | **p-value** | **Phenotype** | **Correlation** | **p-value** |
|  |  |  | % mDC (% of PBMCs) | -0.272 | 0.0006 |
| **% CD28- CD4+ T cells** | | | | | |
| **Phenotype** | **Correlation** | **p-value** | **Phenotype** | **Correlation** | **p-value** |
| % NK T cells (% of PBMCs) | 0.554 | 9.54E-14 | CD4+ cells (% of CD3+) | -0.268 | 0.0008 |
| CD3+ cells (% of Live) | 0.271 | 0.0007 | % NK cells (% of PBMCs) | -0.286 | 0.0003 |
| **% CD28- CD8+ T cells** | | | | | |
| **Phenotype** | **Correlation** | **p-value** | **Phenotype** | **Correlation** | **p-value** |
| Naive CD8+ cells (% of CD8+) | 0.401 | 2.56E-07 | Treg memory (% of Treg) | -0.266 | 0.0008 |
| % NK T cells | 0.396 | 3.75E-07 |  |  |  |
| CD8+ (% of CD3+) | 0.269 | 0.0007 |  |  |  |
| **CD4/CD8 ratio** | | | | | |
| **Phenotype** | **Correlation** | **p-value** | **Phenotype** | **Correlation** | **p-value** |
| CD4+ cells (% of CD3+) | 0.936 | 1.15E-70 | CD8+ (% of CD3+) | -0.968 | 5.81E-93 |
|  |  |  | CD127+ memory CD4 T cells (% of CD4+) | -0.345 | 1.20E-05 |
|  |  |  | % NK T Cells | -0.322 | 4.60E-05 |

Abbreviations: HLA = HLA-DR, mDC = myeloid dendritic cell, pDC = plasmacytoid dendritic cell, Treg = regulatory CD4+ T cell.
