# Supplementary figures and images for: Immunosenescence-Related Transcriptomic and Immunologic Changes in Older Individuals Following Influenza Vaccination
Source: Front Immunol. 2016 Nov 2;7:450. doi: 10.3389/fimmu.2016.00450 (PMC5089977; doi:10.3389/fimmu.2016.00450)

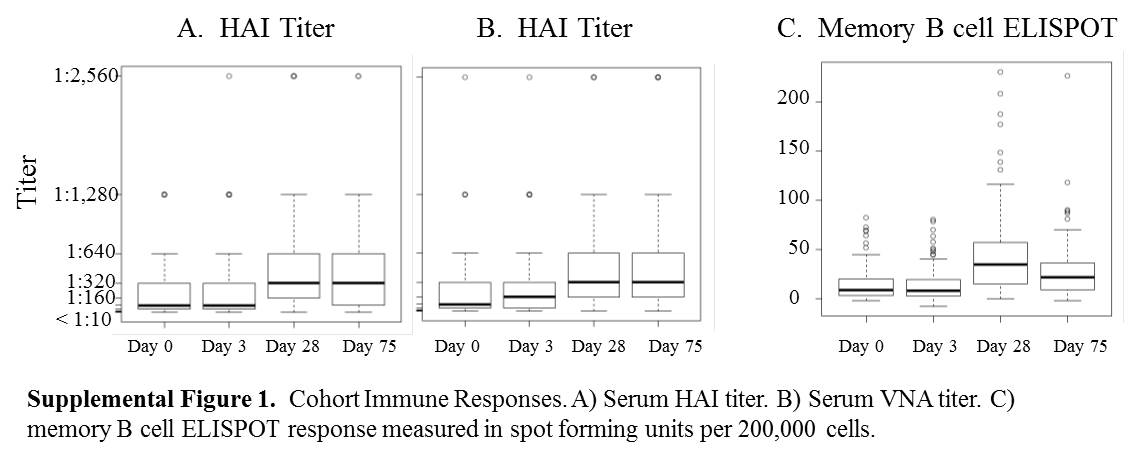

Supplement: Supplementary file 8 [file Image_1.JPEG]

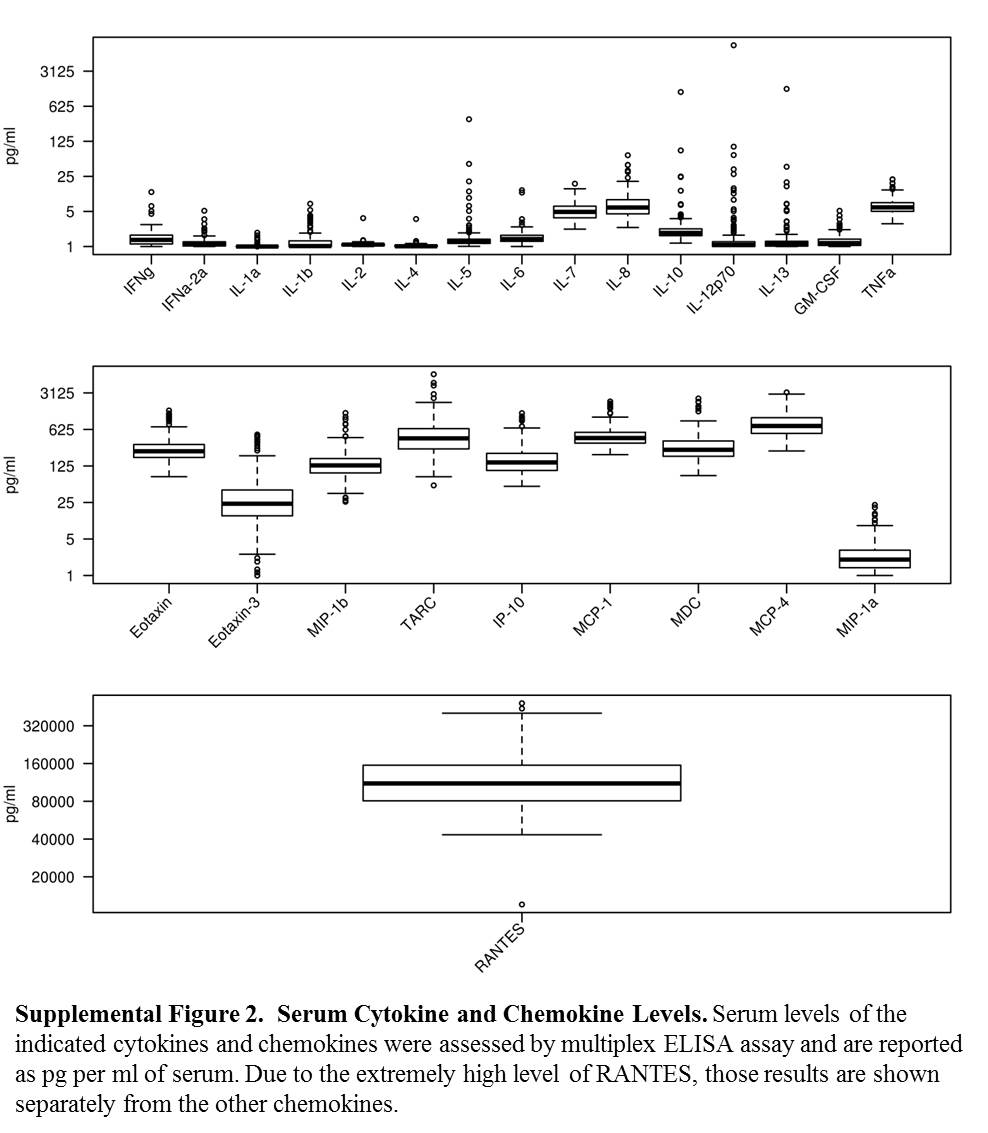

Supplement: Supplementary file 9 [file Image_2.JPEG]
